# AlphaFold Meets De Novo Drug Design: Leveraging Structural Protein Information in Multitarget Molecular Generative Models

**DOI:** 10.1021/acs.jcim.4c00309

**Published:** 2024-10-30

**Authors:** Andrius Bernatavicius, Martin Šícho, Antonius P. A. Janssen, Alan Kai Hassen, Mike Preuss, Gerard J. P. van Westen

**Affiliations:** † Leiden Academic Centre for Drug Research, Leiden University, Einsteinweg 55, 2333CC Leiden, The Netherlands; ‡ Leiden Institute of Advanced Computer Science, Leiden University, Niels Bohrweg 1, 2333CA Leiden, The Netherlands; ¶ CZ-OPENSCREEN: National Infrastructure for Chemical Biology, Department of Informatics and Chemistry, Faculty of Chemical Technology, University of Chemistry and Technology Prague, Technická 5, 166 28 Prague, Czech Republic; § Leiden Institute of Chemistry, Leiden University, Einsteinweg 55, 2333CC Leiden, The Netherlands

## Abstract

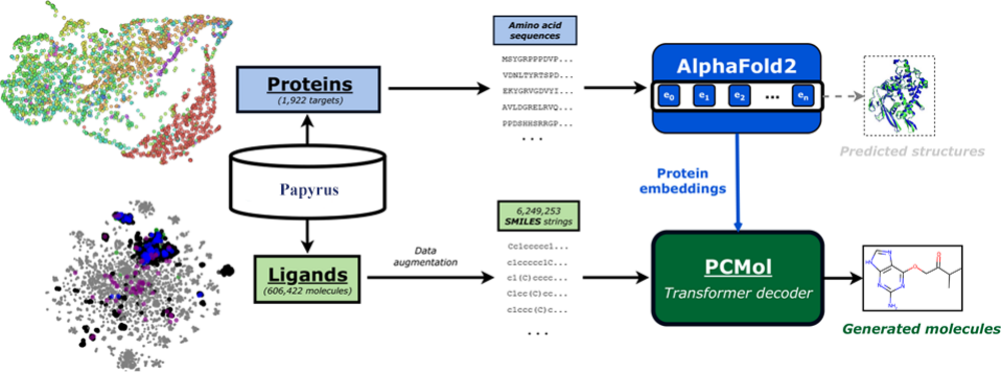

Recent advancements in deep learning and generative models have significantly expanded the applications of virtual screening for drug-like compounds. Here, we introduce a multitarget transformer model, *PCMol*, that leverages the latent protein embeddings derived from *AlphaFold2* as a means of conditioning a de novo generative model on different targets. Incorporating rich protein representations allows the model to capture their structural relationships, enabling the chemical space interpolation of active compounds and target-side generalization to new proteins based on embedding similarities. In this work, we benchmark against other existing target-conditioned transformer models to illustrate the validity of using AlphaFold protein representations over raw amino acid sequences. We show that low-dimensional projections of these protein embeddings cluster appropriately based on target families and that model performance declines when these representations are intentionally corrupted. We also show that the *PCMol* model generates diverse, potentially active molecules for a wide array of proteins, including those with sparse ligand bioactivity data. The generated compounds display higher similarity known active ligands of held-out targets and have comparable molecular docking scores while maintaining novelty. Additionally, we demonstrate the important role of data augmentation in bolstering the performance of generative models in low-data regimes. Software package and AlphaFold protein embeddings are freely available at https://github.com/CDDLeiden/PCMol.

## Introduction

In order to reduce the substantial financial and time investments associated with drug development, research efforts are increasingly incorporating computational techniques as a means of identifying new candidate compounds in silico.^1^ In vitro, the presence of drug-target interactions that are necessary for binding can be verified by measuring the binding affinity using various bioactivity assays.^2,3^ However, this costly process is often preceded by extensive virtual screening of molecules, for example through the usage of physics-based computational methods such as molecular docking or molecular dynamics (MD)^4^ as well as Quantitative Structure–Activity Relation (QSAR) models^5^ that can coarsely predict these desirable interactions. Virtual screening typically involves selecting candidate compounds from large libraries of synthesizable molecules such as Enamine Real^6^ and ZINC^7^ or, alternatively, generating them from scratch using de novo molecule generation models.

Given the vastness of the space of possible drug-like compounds, estimated to be around 10^60^,^8^ the purpose of these generative models is often 2-fold. They need to have the capacity to exploit the known active compound space contained within ligand bioactivity data sets such as CHEMBL^9^ or Papyrus,^10^ while also being flexible enough to propose novel chemical structures that satisfy particular desirable molecular property constraints. To achieve this, state-of-the-art de novo drug design approaches rely on a wide array of generative machine learning techniques,^11^ such as reinforcement learning (RL),^12,13^ generative adversarial networks,^14,15^ variational autoencoders,^16,17^ autoregressive models such as recurrent neural networks^18^ or transformers,^19,20^ and more recently - diffusion models.^21,22^

Within their optimization routines, these de novo generative models often make use of other predictive models that can estimate specific desirable molecular characteristics. In particular, these approaches frequently rely on Quantitative Structure–Activity Relation (QSAR) models in order to score and rank the generated compounds. These models use various numerical descriptors for representing molecules that help estimate the binding affinity to a specific target protein.^23^ QSAR models can be extended by incorporating target information in the form of protein embeddings - the technique referred to as proteochemometrics (PCM),^24^ or drug-target interaction modeling (DTI).^25^ The ability to predict the bioactivity of a ligand on multiple protein targets increases the applicability range of these models and can potentially identify off-target effects or identify highly selective ligands.^24^ Additionally, this enables the use of larger training data sets, which is a highly desirable feature in deep learning applications.^26^

In a fashion similar to QSAR models, traditional de novo molecule generative approaches are usually tailored to generate compounds for a single target or a restricted subset of homologous proteins.^27,28^ However, more recent methods are beginning to incorporate additional structural constraints based on the target of interest to steer the molecular generation process, offering benefits akin to PCM modeling described earlier. One category of such models directly uses the amino acid sequence of the target protein for conditioning.^29−31^ However, given the complex relationship between an amino acid sequence and the corresponding 3D shape of the protein, it can be argued that the sequence alone lacks sufficient information to accurately describe the properties of protein targets without additional preprocessing or modeling. Alternatively, other approaches employ more localized structural target representations that focus on the active site of the protein, where both the ligand and the binding pocket are represented as interacting graphs.^32,33^ Diffusion-based generative models are moving this trend forward and showing that viable ligands can be directly generated in 3D within the binding pocket; however, the poses that they generate are often not feasible.^34,35^ Nevertheless, there are still many unexplored possibilities for representing target-based constraints and combining them with ligand-based generative models, making it an intriguing area for research.

The advent and rapid adoption of large protein-language models such as AlphaFold,^36^ ESM,^37^ or RosettaFOLD2^38^ enabled numerous novel developments in the field of computational structural biology and chemistry.^39^ The quality of the predictions generated by these models suggests that the latent protein representations that are extracted by training end-to-end on large corpora of amino acid sequences and 3D protein backbone pairs are particularly rich in their informational content. Additionally, given the scale of computational and financial resources involved in training or reproducing these large-scale models, it is sensible to reuse their learned high-quality protein representations for other downstream tasks.^40^ Several recent studies have explored the validity of this idea by using these AlphaFold embeddings as inputs for other deep learning models. The list of such applications includes molecular docking,^41^ protein binding site prediction,^42^ prediction of conserved or variant viral protein residues,^43^ and protein homology inference.^44^

Here, we introduce a generative model, *PCMol*, which extends the use of latent protein representations of AlphaFold2 to condition a de novo generative transformer model on target protein structures.

## Methods

### Protein Representations

The data for training the proposed generative model consist of two modalities: ligand data and protein representations. Protein targets were selected based on the following criteria: **1)** the sequences were at most 1,536 amino acids in length, and **2)** they had at least 10 active compounds in the publicly available ligand data sets. Proteins from species other than *Homo sapiens* were also included if they met these criteria.

**Figure 1:**
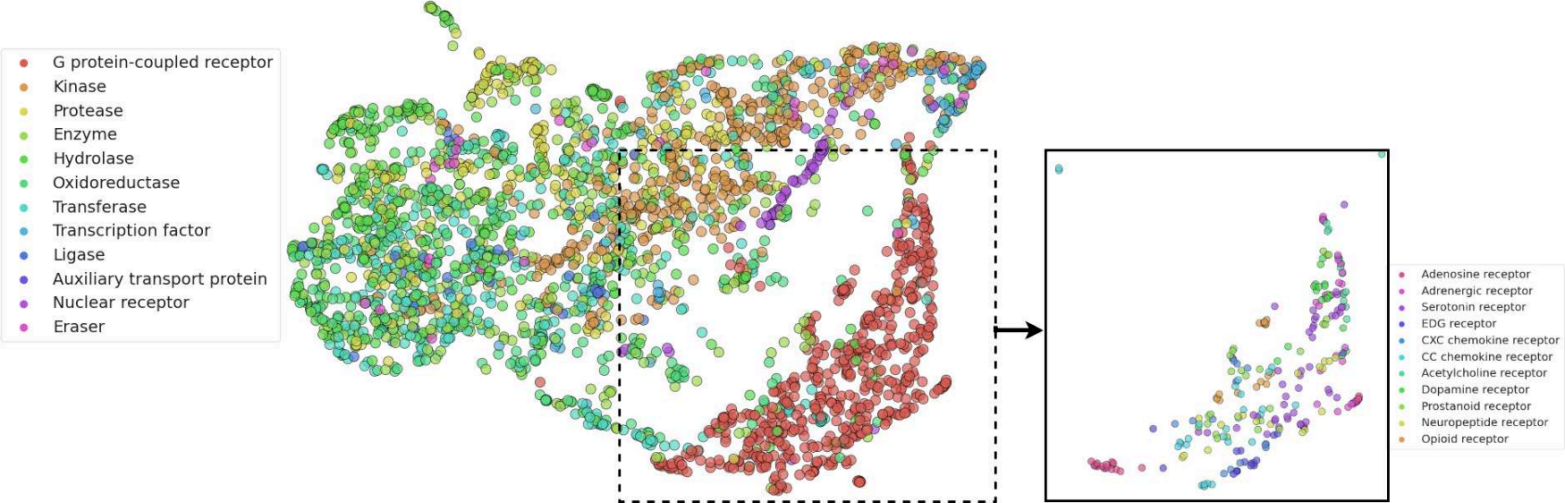
U-MAP of AlphaFold protein embeddings of targets used for training and testing the PCMol model (1990 proteins). A degree of clustering can be seen both at the level of protein families (left) and within the subcategories of the G protein-coupled receptor (GPCR) family (right).

The protein embeddings were obtained by passing the target’s amino acid sequences of selected proteins as input to the AlphaFold model (v2.2.0).^36^ The model was modified so that representations of the target protein following the *Evoformer* and *Structure* modules (embeddings referred to as ***m̃***_***si***_ and ***a***_***i***_ respectively in the AlphaFold Supporting Information)^36^ could be saved and reused during training and inference. It is worth noting that unlike traditional protein descriptors, the size of these embeddings scales based on the length of target amino acid sequences since AlphaFold is based on the transformer architecture. In the case of ***m̃***_***si***_ and ***a***_***i***_ embeddings, each amino acid in the target protein is represented by 384 scalar values, making their tensor size equal to ***m̃***_***si***_ = ***a***_***i***_ = [**384** × ***L***], where ***L*** is the length of the sequence. Due to the property of varying lengths, these embeddings are difficult to visualize using conventional dimensionality reduction techniques without the use of alignment methods. Alternatively, the embeddings of all the protein targets can be cast to an identical shape by averaging over the residue dimension, resulting in vectors of size [**384** × **1**]. The U-MAP^45^ projection of these vectors shown in Figure 1 illustrates a degree clustering between targets based on their protein family or subfamily of these averaged embeddings. Since the PCMol model is reliant on AlphaFold protein representations, a data set of 4,331 processed proteins is included in the repository of this project, which covers a large subset of medium-sized (<1536 residues) biologically relevant protein targets in the ChEMBL^9^ database.

The test set was sampled using a stratified weighted random split based on the target protein families. The weights of the sequences for sampling were determined based on the sequence similarities obtained by sequence alignment using ClustalW.^46^ Functional orthologues from other species were also assigned to the test set to minimize potential data leakage. This resulted in 1,922 targets that were selected for the training set and 68 targets in the test set, covering a wide range of protein families.

### Ligand Data

The molecular data were obtained from the Papyrus bioactivity data set (v5.5),^10^ which encompasses CHEMBL^9^ and ExCAPE-DB^47^ along with several other high-quality data sets and includes standardization of the molecules and assay quality assessments. Molecules were represented using simplified molecular-input line-entry system (SMILES) strings, followed by a tokenization procedure. Stereochemistry information was not used, and only highly active compounds with a *pChEMBL* value ≥ 6.5 (which includes *K*_*i*_, *K*_*d*_, and *IC*_50_ measurements) were selected. This resulted in 661,613 protein–ligand pairs in total (606,422 used for training, 55191 for testing the model).

### Model

**Figure 2:**
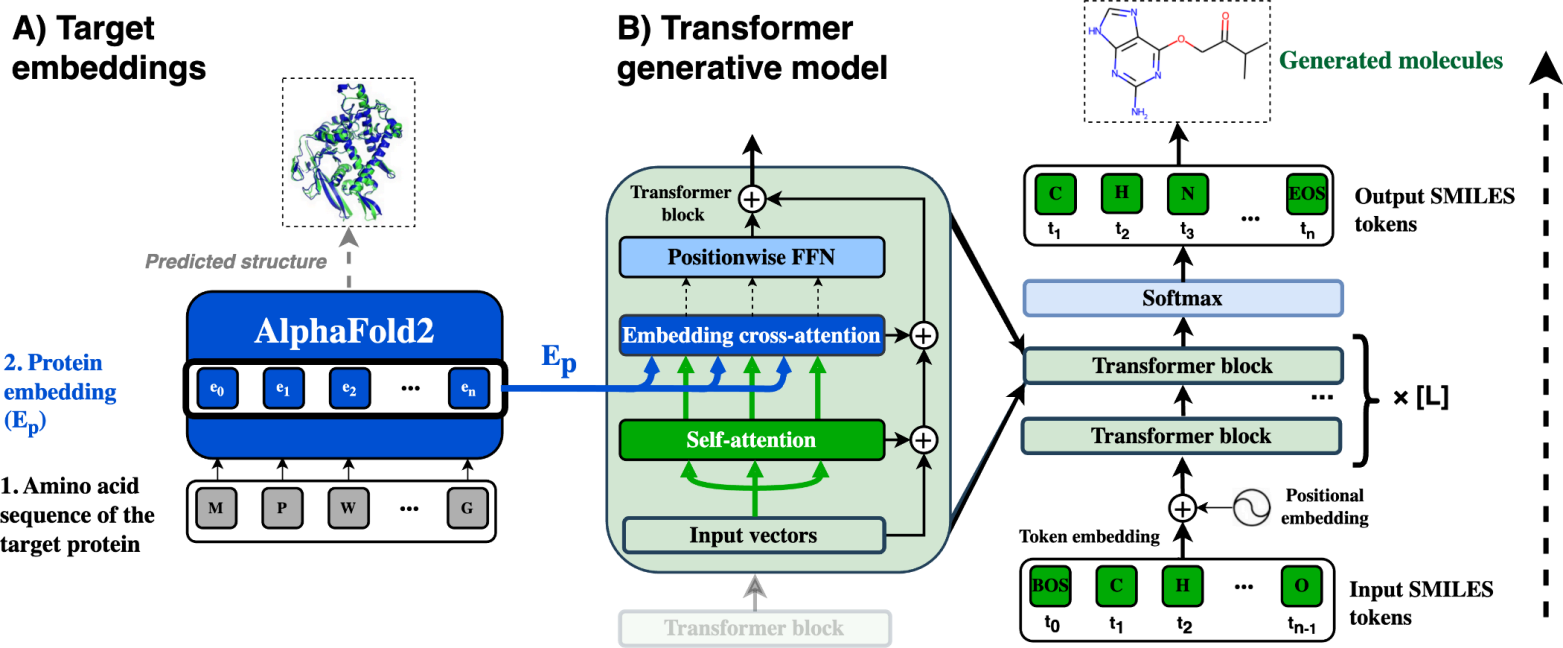
Outline of the PCMol model architecture. A) The protein target embeddings are obtained by running inference on the amino-acid sequence using AlphaFold and saving these embeddings as a data set. B) The embeddings are then used to condition a transformer model to generate SMILES strings for a large set of different targets. The transformer model consists of L identical blocks that use self-attention to process SMILES input vectors and cross-attention for AlphaFold protein embeddings, followed by a position-wise feed-forward network (FFN) layer.

The model is a transformer encoder-decoder^20^ with a cross-attention mechanism to integrate target information from protein embeddings. Therefore, this modeling task can be viewed as a form of translation between the spaces of AlphaFold protein representations and the chemical space of bioactive molecules of specific targets. The model is trained using the regular autoregressive objective, where the task is to predict the next token of a molecule in SMILES string format while being conditioned on an unmasked target protein embedding. The transformer blocks use the Pre-LN^48^ structure, where unlike in the original transformer implementation,^20^ layer normalization precedes the attention layers. Dropout layers were used before the positionwise feed-forward layers in each of the transformer blocks. Transformer positional embeddings were learned as part of the model’s trainable parameters. AlphaFold embeddings of size (***m̃***_***si***_ = ***a***_***i***_ = [**384** × ***L***]) are first passed through a fully connected layer in order to match the size of the transformer decoder’s inner feature dimension *d*. The hyperparameter values used in the final model are listed in Table 1, while the overall architecture is summarized in Figure 2.

**Table 1:** Hyperparameter Setup of the PCMol Generative Encoder-Decoder Transformer Model

**Hyperparameter**	**Value**
Feature dimension (d)	768
Transformer blocks	16
Number of heads	32
Encoder context window	1,536
Decoder context window	102
Dropout	0.1
Batch size	96
Learning rate	9.0e-5
Number of parameters	102,340,995

To study how much protein-side information is being used in this model, an additional variant, termed *PCMol-Zero*, was trained using shuffled AlphaFold embeddings. The embeddings were shuffled both on the amino acid dimension (L) which scales with protein length and along the feature dimension [L × 384]). Identical sets of training data, hyperparameters, and training times were used for both *PCMol* and *PCMol-Zero* models.

### Data Augmentation

**Figure 3:**
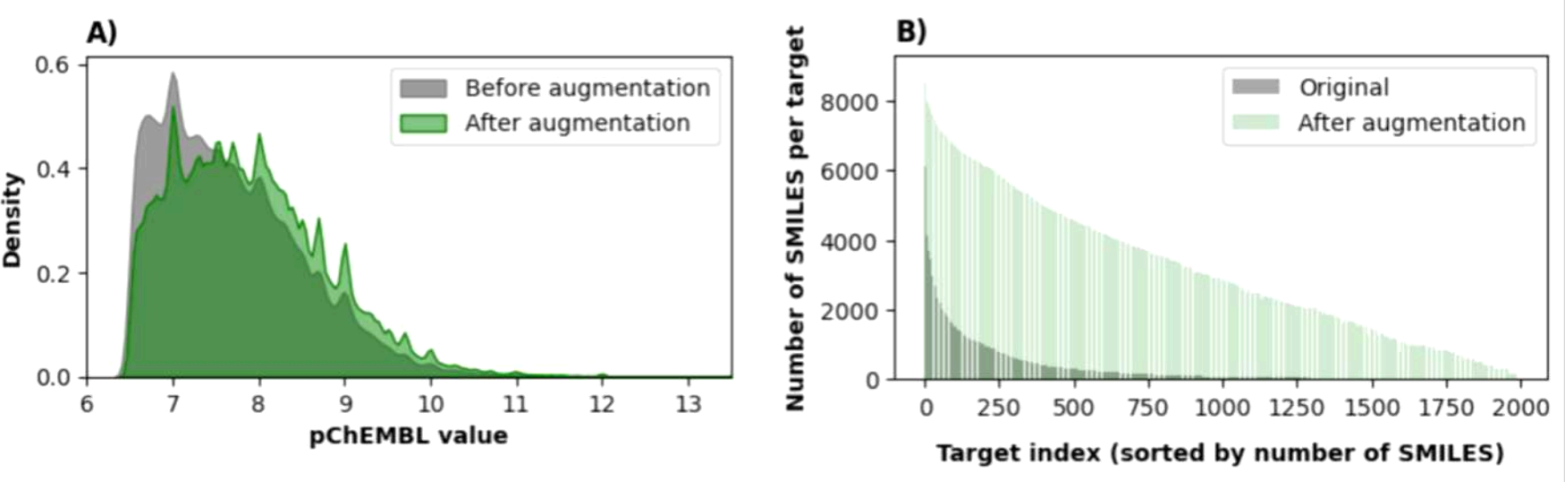
A) The normalized densities of pChEMBL values of molecules in the training set before and after SMILES enumeration due to weighted oversampling based on bioactivity values. B) The change in the number of available SMILES strings in the training data set before and after augmentation. Proteins are sorted along the X axis based on the number of ligands with bioactivity values > 6.5 that are available in the Papyrus data set.

Among the 1,922 proteins that were selected for training, the number of active compounds per target contained in the training data from the Papyrus bioactivity data set varies from 10 to 5,903 and follows a power law distribution where over 72% of the targets have less than a hundred bioactive molecules available, as seen in Figure 3B. If this substantial data imbalance is not accounted for, during training the loss function would steer the model to accurately generate compounds only for the few well-represented protein targets while neglecting the long tail-end of this uneven distribution. This issue is tackled by employing a selective augmentation approach using SMILES enumeration^49^ which utilizes the property that a SMILES string of a particular molecule can be reformulated in a large number of distinct permutations. Usually in generative models this property is either ignored or treated as undesirable as shown by the frequent usage of canonical SMILES or nonvariant Self-Referencing Embedded Strings (SELFIES)^50^ to represent molecules.^51,52^ However, in the case of this multitarget model, this is a favorable property, and augmenting the original SMILES strings achieves the following goals:

1. It scales the number of SMILES strings per protein target in a more linear fashion and lessens the impact of the skewed distribution seen in Figure 3B. The total number of augmentations per target is determined by applying a log transformation, where the new number of ligands per target after augmentation *n*_*i*_*′* is determined by the formula
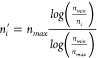
1where *n*_*min*_ and *n*_*max*_ are the minimum and maximum number of available compounds across all targets (10 and 5,903 respectively).
2. Augmentation also shifts the overall distribution of bioactivity values as seen in Figure 3A. Weighted sampling based on the bioactivity value favors highly potent ligands through an increased probability *P*_*j*_ of being selected for augmentation:
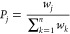
2This probability is scaled by the compound’s weight *w*_*j*_ which depends on its pChEMBL value and scaling constants *r* and *C* that determine the magnitude of selectiveness toward active molecules (equal to 5.5 and 3 respectively).
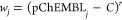
3
3. Additionally, data augmentation also increases the total number of unique protein–ligand SMILES string pairs to be used in training almost 10-fold (661,613 to 6,249,253).

## Results

To illustrate the efficacy of the PCMol model, it is compared to two existing encoder-decoder SMILES generative transformer models – AlphaDrug^31^ and Transformer.^29^ The two latter methods use raw amino-acid sequence strings as the means of conditioning the model on a particular target protein. The goal of the following experiments is to identify whether the use of complex protein embeddings adds value in the generation of novel compounds for a wide range of targets. Additional information on the methodology used to generate molecules using the models can be found in Section 3 of the Supporting Information.

### Similarity to Known Binders

The experiments below are based on comparing the chemical similarities of target-specific generated compounds compared to the known active binders. 100 molecules were generated for each of these proteins, and the Tanimoto similarity was then measured by comparing the ECFP4 fingerprints (1024 bits, radius of 2).^53^

#### Generalization to Unseen Targets

**Figure 4:**
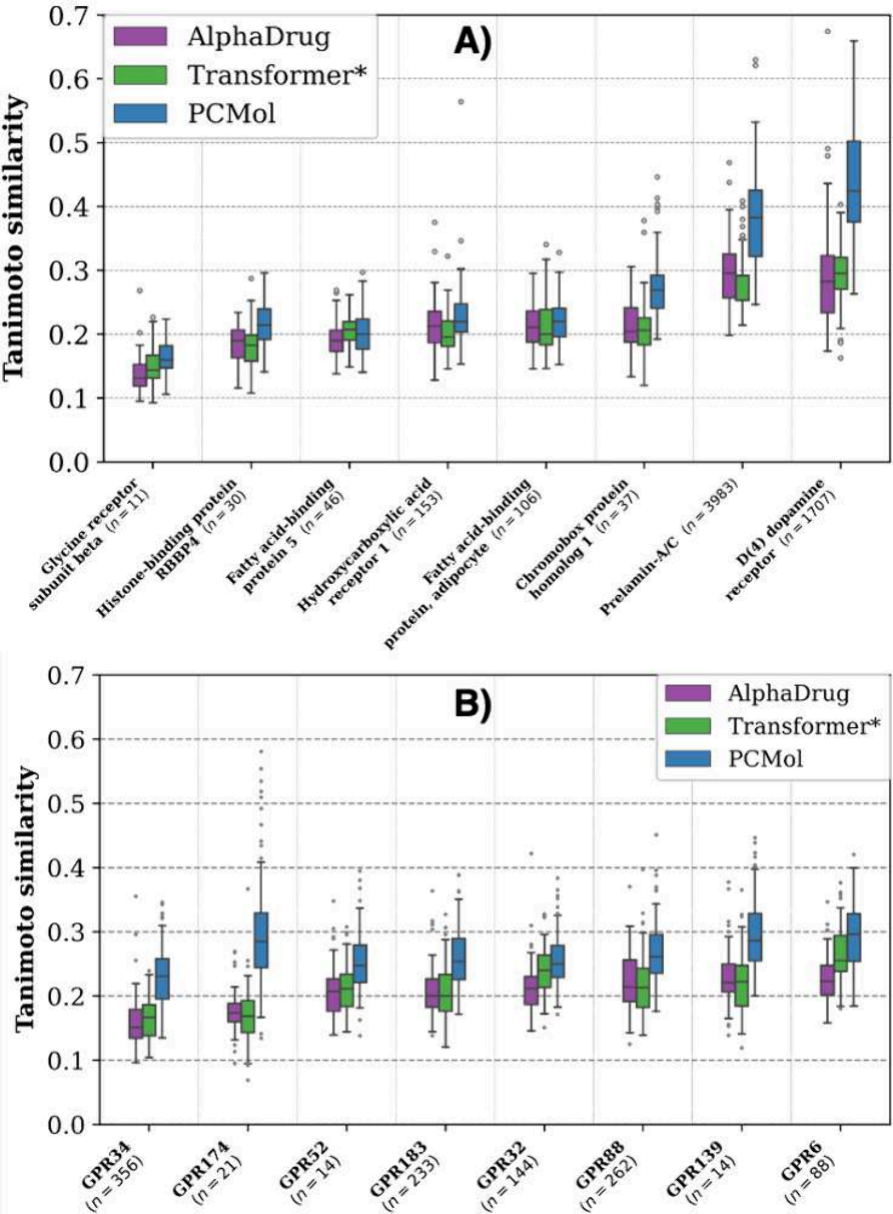
Generalization capability of each model is measured by the distribution of the closest Tanimoto similarities of the 100 molecules generated for each target from the test set. The targets were selected from the intersection of test sets of all 3 models (targets unseen during training). The values ***n*** below the protein target labels denote the number of available active (pChEMBL > 6.5) compounds in the bioactivity databases. **A)** Targets belonging to various protein families. **B)** Recently deorphaned G protein-coupled receptors.

One of the main advantages of target-conditioned models is their ability to generate molecules for targets unseen during training when presented with the associated protein representation. To compare the three methods in terms of their capability to generalize to new targets, a subset of proteins that were not used in the training of either three of these models was determined. These targets had to have at least 10 active ligands (pChEMBL > 6.5) in the existing bioactivity data sets and belong to *Homo sapiens*. This resulted in 19 targets, 8 of which are either orphan or recently deorphaned G protein-coupled receptors.^54^Figure 4 illustrates the distribution of the highest Tanimoto similarities to ligands in the bioactivity data sets of each of the 100 generated molecules per model.

Figure 4 illustrates that molecules generated by PCMol consistently have higher Tanimoto similarity values to known (held-out) ligands compared to the other two methods. However, the generalization performance seems to be varied in some specific cases, particularly Prelamin-A/C and D(4) dopamine receptor, where the similarity values are considerably larger. The better overall performance on these targets could partially be explained by Alphafold embeddings providing more context when compared to raw amino acid sequences as well as a larger overall data set used in training the PCMol model. This is especially useful when generating compounds for targets which have either nonexistent or limited bioactivity data available. This is illustrated by Figure 4B, where the PCMol model is also able to consistently generate molecules that are closer to the unseen data for GPCRs that have until relatively recently been in that target category.

#### Effectiveness of Data Augmentation

**Figure 5:**
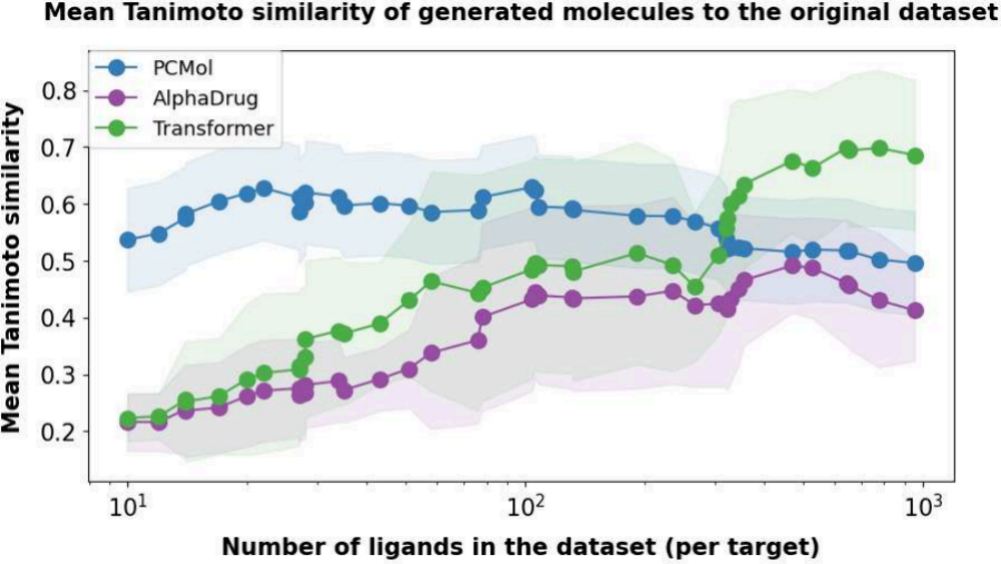
Relationship between the mean Tanimoto similarity of generated compounds and the number of available training data points. Each point represents a randomly selected target from the intersection of training sets of all three generative models.

To test the impact of the data augmentation strategy described earlier, the three generative models were compared in terms of their performance on the targets in the training set; 50 protein targets that all models encountered during the training were selected. The targets were picked in a fashion that covered a varying degree of representation in terms of available active ligands (range of 10–1000 compounds). This selection lets us evaluate the data set memorization capability of each model and measures whether they are capable of producing molecules sufficiently similar to the ones in the training data set when operating in a low-data regime. Since ligand bioactivity data sets are heavily skewed, where a large portion of ligands is associated with only a small fraction of proteins, performance on under-represented targets is a concern from a machine learning perspective. Due to data augmentation, the PCMol model is capable of maintaining relatively stable Tanimoto similarity values of generated molecules on all of the target proteins, regardless of the size of the training subset, ensuring more consistent performance and increasing the applicability range of the model. Figure 5 shows that not using data augmentation often leads to an inability to generate molecules similar to the limited training set. This is particularly true on targets with < 100 available data points which indicates that the models that are not using data augmentation are not always capable of effectively recognizing the target protein sequences as the Tanimoto similarity values of generated compounds are close to those generated for a novel target outside of the training set.

#### Performance on Targets Seen during Training

**Figure 6:**
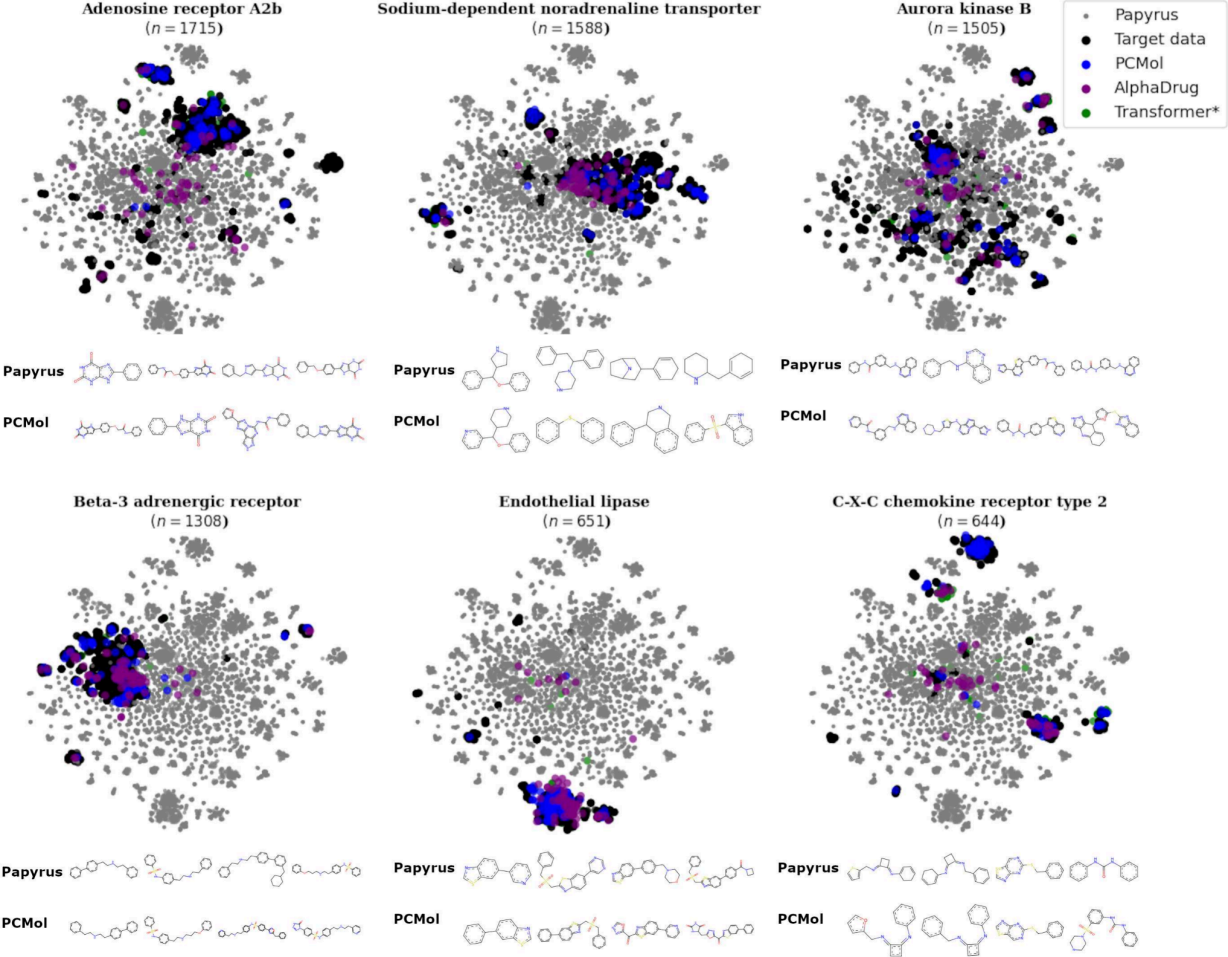
T-SNE plots of the chemical spaces of molecules generated by the generative models for proteins from the training set. Additionally, their respective bioactive ligand sets (black) and the whole training data set (gray) can be seen alongside the 4 most frequently occurring Murcko scaffolds.

Another important performance metric of target-conditioned models is their ability to generate appropriate compounds for a large set of diverse targets. This can be evaluated by looking at how the distributions of generated molecules change, depending on the input embedding or sequence. Figure 6 illustrates this ability by using T-SNE clustering on the fingerprints of generated molecules and comparing them to the known bioactive compounds for that target in the context of the whole training data set. We can see that the generated compounds are highly specific depending on the target and cover diverse parts of the chemical space available in the Papyrus^10^ bioactivity data set. It is also worth noting that regardless of the target chosen, a small portion of molecules generated by all 3 models is clustered around the centroid of the overall chemical space. This suggests that target-conditioned models are prone to occasionally generating molecules that are simply the most likely statistical average of all molecules seen during training, and additional filtering steps could be added to correct for this property.

**Figure 7:**
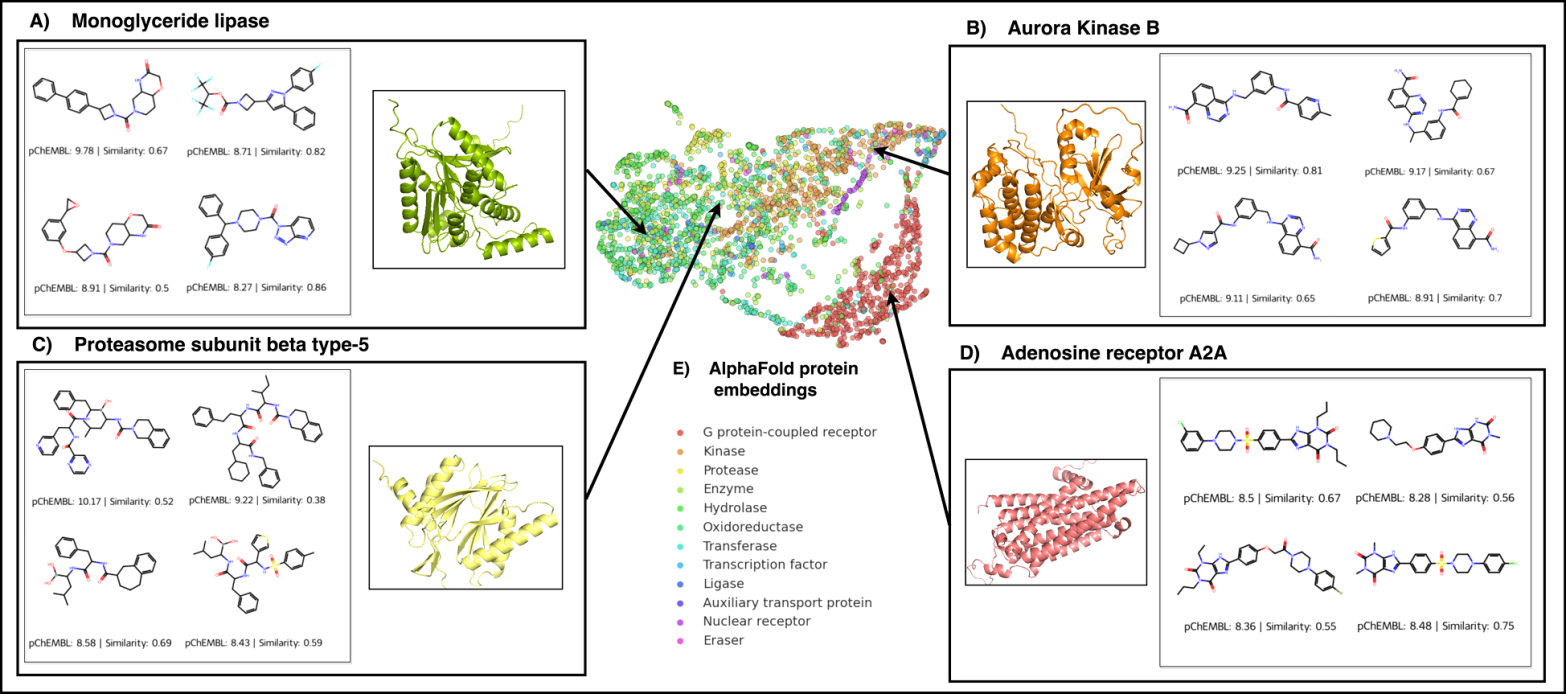
Highest scoring molecules generated for various targets using the PCMol model (compounds selected after generating 100 compounds per target). The predicted pChEMBL values were obtained from individual QSAR models trained on bioactive ligands of that particular target. Tanimoto similarity values of the closest molecule in the training set are also shown alongside each molecule.

Finally, to demonstrate the potential use of the PCMol model, we selected four clinically relevant protein targets for generating new potential ligands. For these targets, individual random forest QSAR models were trained, based on reported ChEMBL ligands (for more information refer to Supporting Information Figure S3). PCMol was used to generate 100 ligands for each of the targets, which were then scored using the relevant QSAR model. For each protein, four high-ranking ligands are displayed in Figure 7. For all targets, it is evident that PCMol was able to generate ligands in the appropriate chemical space: urea- and carbamate-based inhibitors for MAGL (Figure 7A), quinazoline-containing motives for AURKB (Figure 7B), peptide mimics with boronic acid warheads for PSMB5 (Figure 7C), and the xanthine core for A2AR (Figure 7D). Additionally, the overall median potency of the generated molecules, as predicted by the QSAR models, is above the median of the known compounds for those targets.

### Molecular Docking

Even though it is well-established that molecular docking scores often do not reflect the actual binding affinity of the ligand as the correlation is weak, docking can still be used as a means of selecting more promising compounds when used at a large scale.^55,56^ When statistically compared to scores of known binders, compounds with low scores usually reflect some kind of incompatibility with the target’s binding site and therefore can be filtered out.^57,58^ Additionally, the usage of ligand-protein interaction fingerprints can further improve in-silico compound selection as the presence of desirable interactions with key target residues can lead to higher hit rates.^59^

To verify the viability of the generated compounds, we conducted a virtual screening using molecular docking on 20 targets - 10 from the overlap between training sets and 10 from the test sets (between the PCMol, AlphaDrug,^31^ and Transformer^29^ models). The molecular docking was carried out using *VinaGPU (v2.0)*.^60^ The ligands were then standardized by removing salts, hydrogens, and metal atoms, following the same procedure as described in the Papyrus^10^ data set.

For each of the targets, 100 molecules were generated per model, and the minimum score of all poses was used. Decoys were selected by randomly sampling active ligands of other protein targets from the Papyrus data set. A detailed list of proteins, PDB structure files, and binding site coordinates along with docking score distributions for all individual targets can be found in Table S2 and Figure S4 of the Supporting Information.

**Figure 8:**
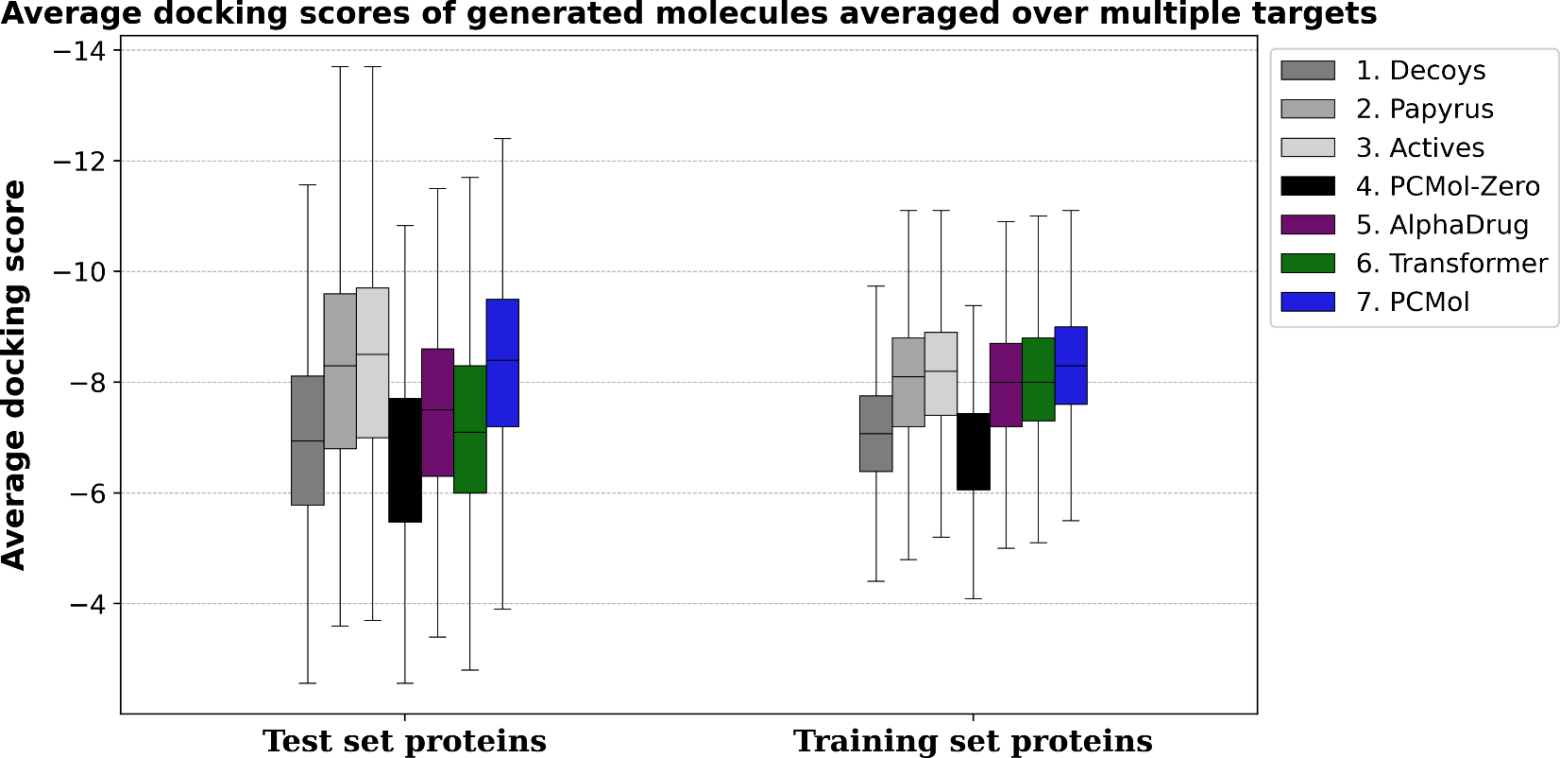
Distributions of average docking scores over 10 test set proteins (left) and 11 training set proteins (right). For each of the targets, 100 compounds were generated and docked per model. Group descriptions: **1) Decoys:** compounds randomly selected from ChEMBL; **2) Papyrus**: randomly selected target-specific compounds; **3) Actives:** target-specific compounds with (pChEMBL ≥ 6.5); **4–7):** Compounds generated by individual models.

Figure 8 summarizes the results, where docking scores are averaged over 11 and 10 proteins for training and test sets respectively. We can see that among real molecules, compounds sampled from the whole Papyrus data set (*Decoys*) consistently have the lowest docking scores. No significant difference is seen between randomly sampled target-specific compounds (*Papyrus*) and compounds with *pChEMBL* ≥ *6.5* (*Actives*). As for synthetic molecules, the ones generated by the *PCMol-Zero* model (a model that uses shuffled AlphaFold embeddings) have scores lower than randomly sampled decoys. As for the three other generative models, molecules generated by PCMol show better docking scores overall, particularly for the proteins unseen during training.

## Discussion and Conclusion

Target-conditioned models offer several specific benefits over traditional de novo generative models that focus on optimizing the performance on individual targets. One great feature is the ability to generate compounds for a diverse set of targets, including ones that are novel or understudied. The improved generalization capabilities to unseen targets of the PCMol model are likely to be a result of multiple contributing factors. AlphaFold protein representations offer rich additional structural protein information, and using them does not require a specialized protein sequence encoder model that needs to be trained when using raw amino acid sequences as input. The *PCMol-Zero* model which was trained using shuffled AlphaFold embeddings is no longer able to generate appropriate ligands even for targets seen using training, let alone novel targets. This indicates that protein-side information is a key factor for the performance of these multitarget generative models. However, one obvious drawback of this approach is that the model can only run when presented with an appropriate AlphaFold embedding, which restricts its use on targets outside of this model’s data set without running additional inference cycles.

Differences in the training data set creation could also have accounted for some of the improvements, both on the target and ligand side. The data augmentation strategy proved to be essential for making the model perform well on targets that have limited bioactivity data. Upsampling highly active ligands resulted in the model generating compounds that have 1) higher similarity to known actives, 2) higher docking scores, and 3) higher bioactivity values predicted by QSAR models. Additionally, as illustrated in numerous recent papers in the deep learning domain, scaling up model and data set sizes does lead to increased sample efficiency and performance.^26^ This trend was observed during the model training phase when larger models and data sets yielded better generalization to the held-out validation sets of unseen targets.

In follow-up work, we are exploring coupling rich 3D molecule representations (either voxel- or graph-based) with the AlphaFold protein representations. Furthermore, a detailed virtual and laboratory screening for targets that a) have no bioactivity data available and b) are close in the AlphaFold embedding space to targets within the training set would be the next step in validating the generalization capabilities of the model.

## Data Availability

The code for running the model along with pretrained model weights is available at the following repository: https://github.com/CDDLeiden/PCMol. The full data set of precomputed AlphaFold protein embeddings is available on Zenodo (*10.5281/zenodo.10671261*). Pretrained weights of the PCMol model can be downloaded from (*10.5281/zenodo.10512870*).

## Supporting Information Available

S1 - Model, S2 - Data set, S3 - Benchmarking, S4 - QSAR modeling, S5 - Molecular Docking (PDF)
