# Supplementary material for: AlphaFold Meets De Novo Drug Design: Leveraging Structural Protein Information in Multitarget Molecular Generative Models
Source: J Chem Inf Model. 2024 Oct 30;64(21):8113–22. doi: 10.1021/acs.jcim.4c00309 (PMC11558674; doi:10.1021/acs.jcim.4c00309)
Supplement: Supplementary file 1 — ci4c00309_si_001.pdf [file ci4c00309_si_001.pdf]

AlphaFold meets de novo drug design: leveraging structural protein information in multi-target molecular generative models

Supporting information

Andrius Bernatavicius,[†,‡] Martin Šícho,[†,¶] Antonius P.A. Janssen,[†,§] Alan Kai Hassen,[‡] Mike Preuss,[‡] and Gerard J.P. van Westen[*,†]

†*Leiden Academic Centre for Drug Research, Leiden University, Einsteinweg 55, 2333CC Leiden, The Netherlands*

‡*Leiden Institute of Advanced Computer Science, Leiden University, Niels Bohrweg 1, 2333CA Leiden, the Netherlands*

¶*CZ-OPENSCREEN: National Infrastructure for Chemical Biology, Department of Informatics and Chemistry, Faculty of Chemical Technology, University of Chemistry and Technology Prague, Technická 5, 166 28, Prague, Czech Republic*

§*Leiden Institute of Chemistry, Leiden University, Einsteinweg 55, 2333CC Leiden, The Netherlands*

1. Model

The model uses a transformer encoder-decoder architecture often used in language translation tasks. Transformer blocks use the pre-norm ordering, implemented in *PyTorch*. The model was trained on a server with 4x NVidia A30 GPUs (96GB VRAM total) for 438,391 batches of size 96 (around 7 days of runtime).

Table S1: Hyperparameter setup of the PCMol generative encoder-decoder transformer model.

**Hyperparameter**	**Value**
Inner dimensionality (d)	768
Transformer blocks	16
Number of heads	32
Encoder context window	1,536
Decoder context window	102
Dropout	0.1
Batch size	96
Learning rate	9.0e-5
Number of parameters	102,340,995

2. Dataset

The time complexity of transformer models scales quadratically based on the size of the context window. Therefore, to constrain the training/inference time of the model, the optimal values for the length cutoffs for both protein sequences and SMILES had to be determined. This value was chosen so that a significant portion of the available data would still be contained in the training set. In the case of protein sequences, our goal was to retain around 95% of the proteins from the Papyrus dataset which satisfied the other criterion of having at least 10 active (¿6.5 pChEMBL) compounds in the dataset. A cutoff point of 1,536 was chosen as it is a composite of 2 numbers of powers of two (1024 + 512).

The cutoff for the number of tokens of the SMILES strings was chosen at 102 as this enabled the use of more than 99% of SMILES contained in the dataset (Figure S2).

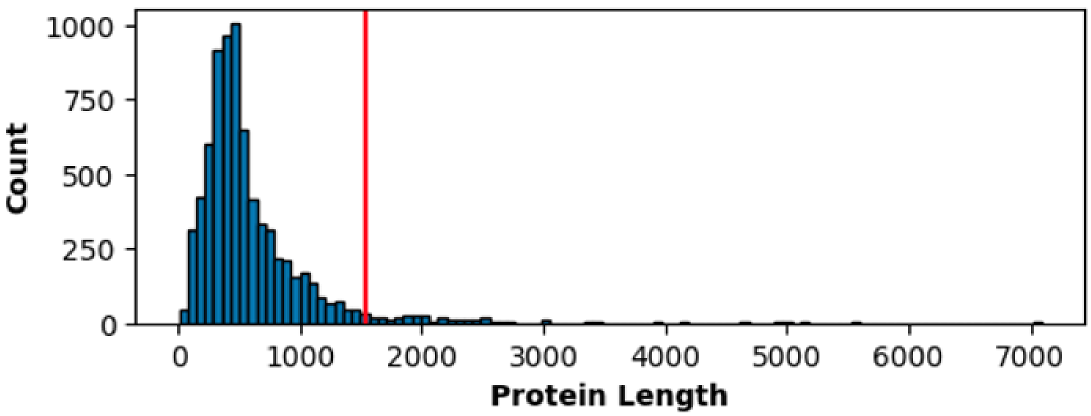

Figure S1: *Protein length cutoff chosen for the training dataset.*

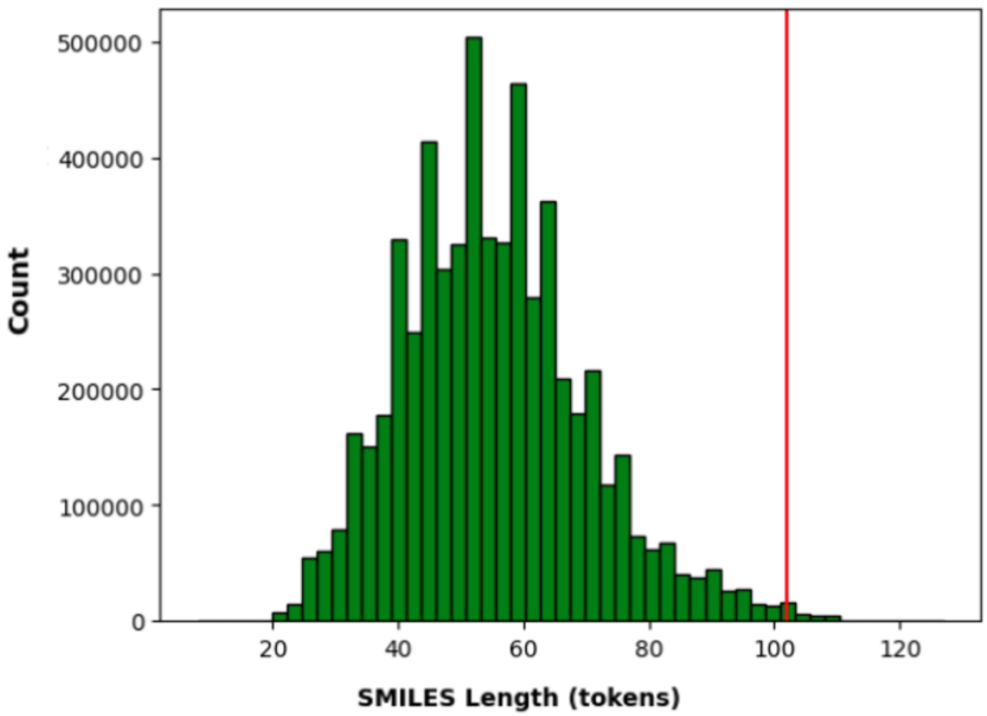

Figure S2: *Cutoff for the number of tokens in a SMILES string used in the training set.*

3. Benchmarking

The performance of the PCMol model is compared to two other models – AlphaDrug and Transformer. The protocol that was used to generate molecules using these models is described below. 100 molecules were generated for each target during evaluation.

PCMol model had two distinct variants, each utilizing different kinds of protein representations:

- **PCMol:** using regular internal Alphafold embeddings to represent proteins.
- **PCMol-Zero:** using shuffled embeddings, where AlphaFold representations are shuffled both on the amino acid dimension (L) which scales with protein length and along the feature dimension $[L \times 384]$).

AlphaDrug

The molecules were generated using the model weights provided by the authors by using the beam search script. Specifically, the **LT** variant of AlphaDrug model was used as it reportedly had the best overall performance. The MCTS functionality was not used.

Transformer

As the original repository did not provide the weights of a trained model, it was retrained from scratch using the hyperparameters provided in the publication by using the Jupyter notebooks in the project's repository. The molecules for each target were then generated using the provided notebook by using the amino acid sequence of individual proteins as input.

4. QSAR Modelling

QSAR models used in evaluating generated molecules were trained using the QSPRpred Python package. Models were trained using ECFP4 fingerprints (bits=1024, radius=2) as features.

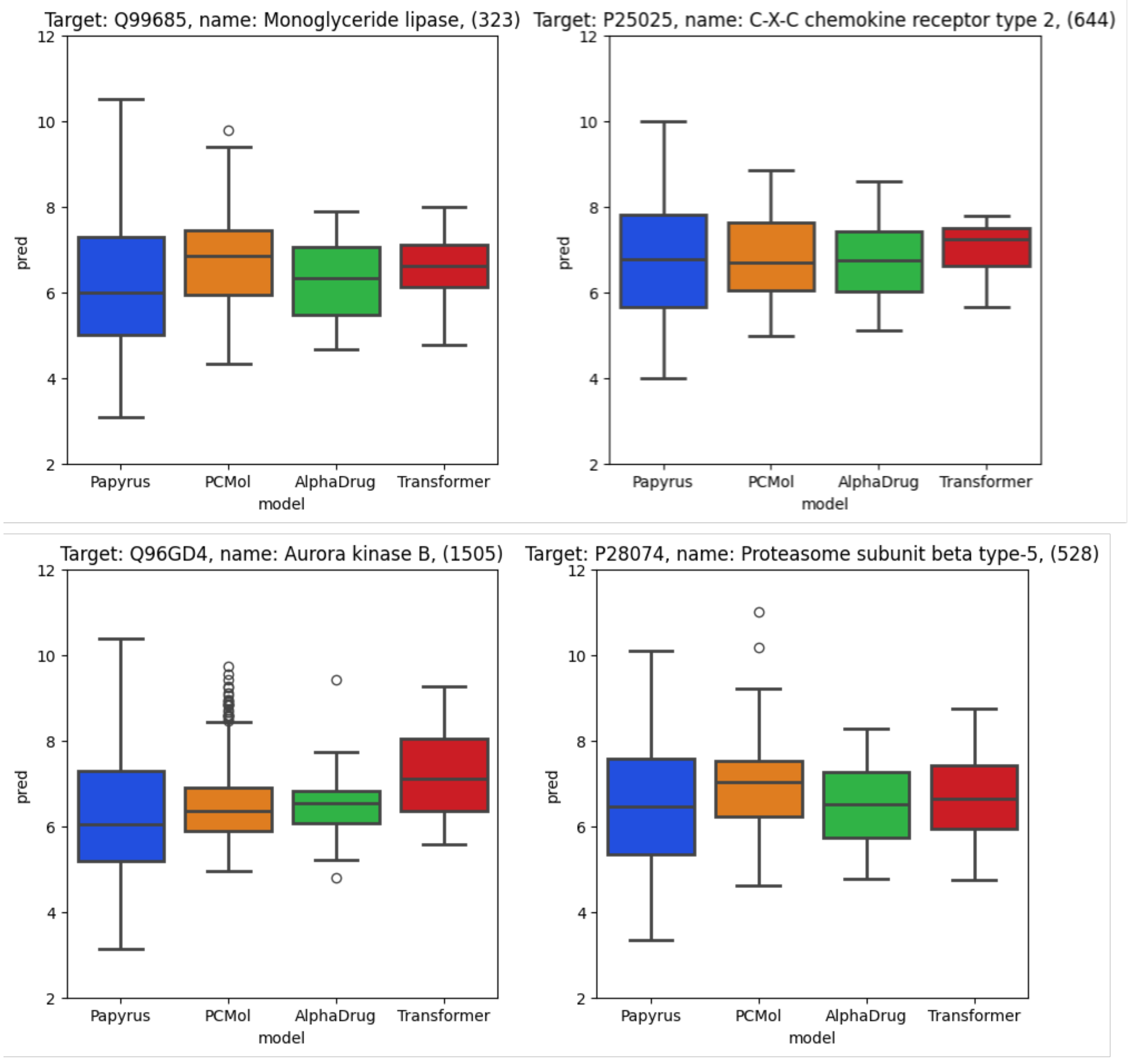

Figure S3: *Distributions of predicted pChEMBL values of 100 compounds generated by different generative models. The range of pChEMBL values of the ligands in the bioactivity datasets is also illustrated as the first bar of each plot. The number of bioactive compounds used in QSAR model training is denoted in the title of each subplot (after the name of the target protein in brackets). All QSAR models had* r^2 *values* ≥ 0.6 *as evaluated by testing on a 75%/25% random split.*

5. Molecular docking

The molecular docking study was carried out using *VinaGPU (v2.0)*. Ligands were prepared using the *meeko* Python package.

Table S2: *A list of protein targets used in the molecular docking study. The PDB files were selected on the basis of 1) presence of a co-crystallized ligand 2) recency. The binding site coordinates (columns x, y, z) were obtained by calculating the centroid of the ligand and then removing it.*

**Name**	**Uniprot**	**PDB**	**x**	**y**	**z**	**Training set**
Casein kinase I isoform epsilon	P49674	4HNI	-27	-16	-29	0
Oxidized purine nucleoside triphosphate hydrolase	P36639	5FSI	10	21	3	0
D(4) dopamine receptor	P21917	5WIU	-17	14	-17	0
Histone-binding protein RBBP4	Q09028	7M40	2	3	14	0
Protein-serine O-palmitoleoyltransferase porcupine	Q9H237	7URC	113	106	109	0
Chromobox protein homolog 1	P83916	6DO7	13	13	13	0
Proto-oncogene tyrosine-protein kinase Src	P12931	1O45	19	21	22	0
GPR88	Q9GZN0	7WZ4	129	123	172	0
GPR139	Q6DWJ6	7VUG	128	143	145	0
GPR52	Q9Y2T5	6LI0	34	71	66	0
Cyclin-dependent kinase 2	Q9Y5N1	1AQ1	2	28	10	1
Mitogen-activated protein kinase 1	P28482	1PME	-13	13	41	1
Histamine H3 receptor	Q9Y5N1	7F61	-20	51	2	1
Mu-type opioid receptor	P35372	8EF5	101	106	128	1
Adenosine receptor A2b	P29275	7XY6	124	131	150	1
Aurora kinase B	Q96GD4	4AF3	22	-22	-9	1
Beta-3 adrenergic receptor	P13945	9IJD	121	124	86	1
Sodium-dependent noradrenaline transporter	P23975	8WTY	102	112	109	1
C-X-C chemokine receptor type 2	P25025	6LFL	52	-35	190	1
Cyclin-dependent kinase 8	P49336	5IDN	-8	10	-7	1
Monoglyceride lipase	Q99685	8AQF	131	18	-7	1
Casein kinase I isoform alpha	P48729	6GZD	-4	-17	-7	1
Cytochrome P450 1A1	P04798	6UDL	45	34	9	1

Table S3: Hyperparameters used with *VinaGPU (v.2.0)*.

**Hyperparameter**	**Value**
Search depth	9
Threads	2048
Threads per call	512
Bounding box size	(20, 20, 20)

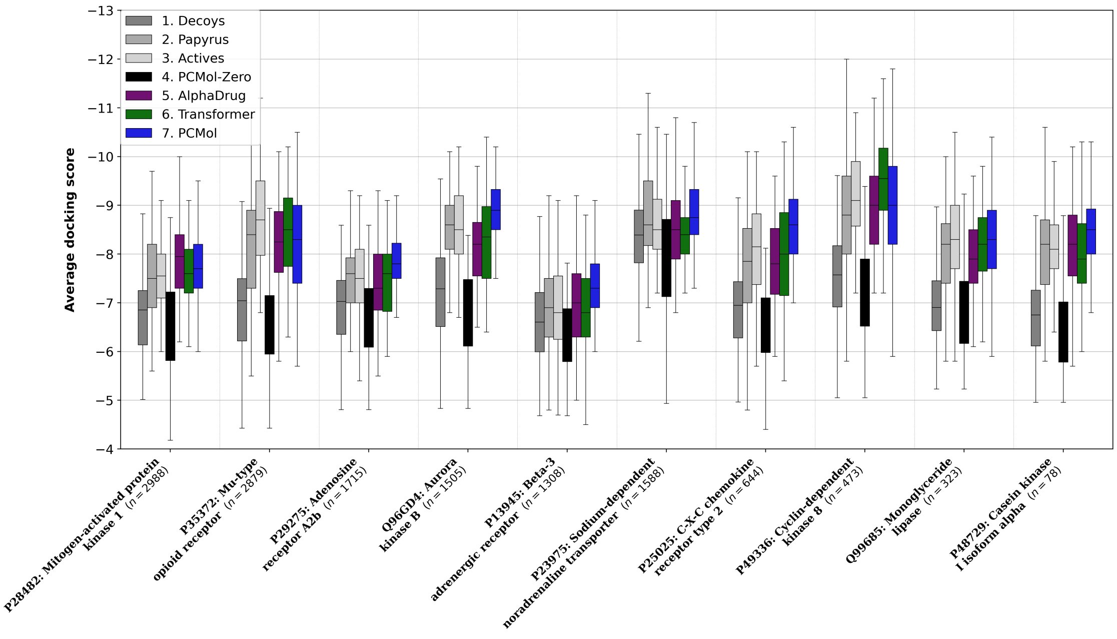

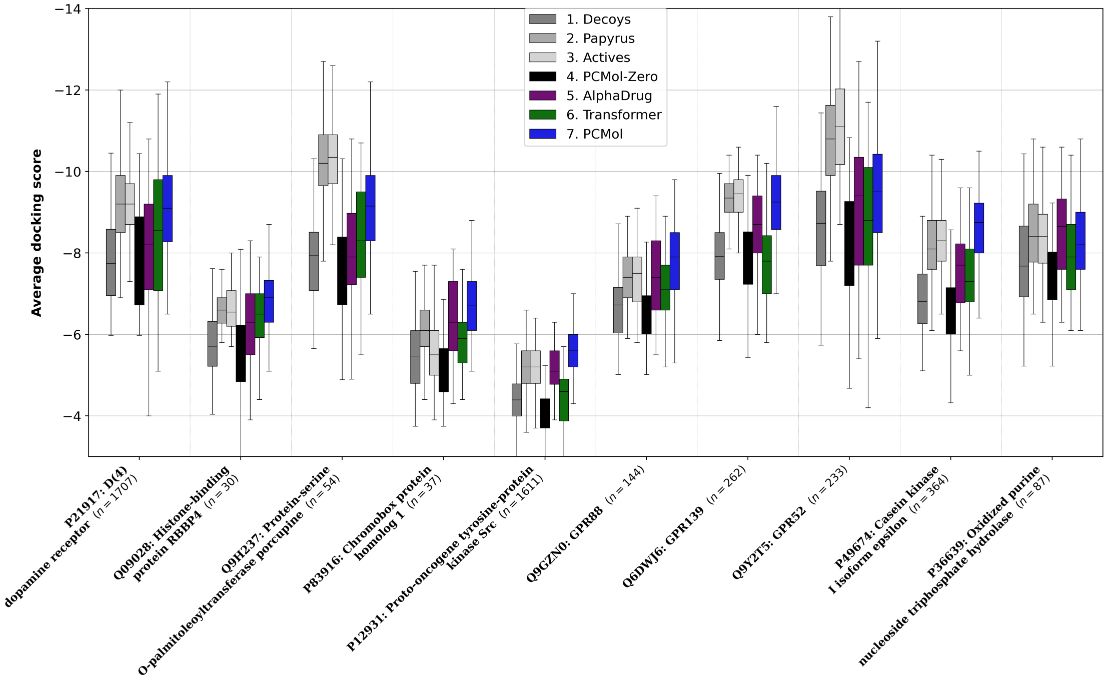

Figure S4: *Distributions of docking scores of 10 training set proteins (Top) and 10 test set proteins (Bottom). For each of the targets, 100 compounds were generated and docked per model, and the minimum score of the top pose was measured. Group descriptions:* ***1) Decoys:*** *compounds randomly selected from ChEMBL;* ***2) Papyrus****: randomly selected target-specific compounds;* ***3) Actives:*** *target-specific compounds with (pChEMBL* $\geq$ *6.5);* ***4-7):*** *Compounds generated by individual generative models.*
